# Systemic Bud Induction and Retinoic Acid Signaling Underlie Whole Body Regeneration in the Urochordate Botrylloides leachi

**DOI:** 10.1371/journal.pbio.0050071

**Published:** 2007-03-06

**Authors:** Yuval Rinkevich, Guy Paz, Baruch Rinkevich, Ram Reshef

**Affiliations:** 1 Faculty of Biology, Technion—Israel Institute of Technology, Haifa, Israel; 2 Israel Oceanographic and Limnological Research, National Institute of Oceanography, Haifa, Israel; University of California Berkeley, United States of America

## Abstract

Regeneration in adult chordates is confined to a few model cases and terminates in restoration of restricted tissues and organs. Here, we study the unique phenomenon of whole body regeneration (WBR) in the colonial urochordate Botrylloides leachi in which an entire adult zooid is restored from a miniscule blood vessel fragment. In contrast to all other documented cases, regeneration is induced systemically in blood vessels. Multiple buds appear simultaneously in newly established regeneration niches within vasculature fragments, stemming from composites of pluripotent blood cells and terminating in one functional zooid. We found that retinoic acid (RA) regulates diverse developmental aspects in WBR. The homologue of the RA receptor and a retinaldehyde dehydrogenase-related gene were expressed specifically in blood cells within regeneration niches and throughout bud development. The addition of RA inhibitors as well as RNA interference knockdown experiments resulted in WBR arrest and bud malformations. The administration of all-*trans* RA to blood vessel fragments resulted in doubly accelerated regeneration and multibud formation, leading to restored colonies with multiple zooids. The *Botrylloides* system differs from known regeneration model systems by several fundamental criteria, including epimorphosis without the formation of blastema and the induction of a “multifocal regeneration niche” system. This is also to our knowledge the first documented case of WBR from circulating blood cells that restores not only the soma, but also the germ line. This unique *Botrylloides* WBR process could serve as a new in vivo model system for regeneration, suggesting that RA signaling may have had ancestral roles in body restoration events.

## Author Summary

Whole body regeneration (WBR) in Animalia is rare, confined to morphologically less complex taxa such as sponges, cnidarians, and flatworms. In the chordate phylum, only colonial ascidians (invertebrate chordates also known as sea squirts) have the documented ability to wholly regenerate. Once separated from the colony, any minute fragment of peripheral blood vessel (about 1 mm in length, containing 100–300 blood cells) of the colonial ascidian Botrylloides leachi regenerates an entire functional adult within one to three weeks. By following cellular and molecular events in *Botrylloides* WBR, we revealed that this system proceeds differently from regeneration events in other model organisms by several fundamental criteria. This is, for example, to our knowledge the first documented case of WBR initiating from circulating blood cells that restore not only the body tissue, but also the germ line. We found that retinoic acid (RA) signaling, previously reported in the regeneration of specific vertebrate tissues and organs, plays a major role in WBR via RA receptor expression throughout the entire regenerating animal. This suggests that RA signaling may have had ancestral roles in body restoration events. Elucidating the processes involved in this WBR system will improve our understanding of the nature of regeneration and the reduced regeneration capabilities represented in so many vertebrates.

## Abstract 3

The unique*Botrylloides* whole body regeneration process could serve as a new in vivo model system for regeneration, suggesting that retinoic acid signaling may have had ancestral roles in body restoration events.

## Introduction

Some of the most fundamental issues in developmental biology concern the ability of metazoans to regenerate. In most multicellular organisms, adult stem cells maintain organs' homeostasis throughout life and facilitate tissue repair after injury or disease [1]. Several organisms are capable of regrowing amputated organs and body parts, for example, amphibian limbs, lens, and retina [2–4]. Phylogenetic perspectives reveal a decrease in regenerative abilities concomitant with an increase in animal body complexity [5]. Thus, only a few adult animals manifest massive regeneration events, and these events occur mostly in less complex multicellular organisms such as sponges [6], cnidarians [7], and flat worms [8]. An exception is the phenomenon of whole body regeneration (WBR) in the highly evolved urochordates subfamily, *Botryllinae*. In this group of sedentary colonial organisms, which are the closest living relatives of vertebrates [9], a fully functional adult can regenerate within 10–14 d from any isolated minute fragment (0.1 mm) of a blood vessel containing a limited number (*n* = 100–300) of blood cells [10,11].

The filter-feeding Atlantic urochordate B. leachi (Figure 1A) is a common encrusting colonial sea squirt, probably a Mediterranean species that has spread ubiquitously [12]. Animals are found in very shallow waters, under stones, on algae, pilings, floats, and other substrata. Each colony is composed of several to thousands of genetically identical modules (zooids) (Figure 1A, arrowheads), each 2–3 mm long, embedded within a gelatinous matrix called the tunic. Zooids are arranged in systems of two parallel elongated and often serpentine rows. A network of blood vessels connects all zooids within a colony, from which pear-shaped vascular termini (ampullae) extend toward the colony margins (Figure 1A, arrows). Once the planktonic larva metamorphoses into the founder individual (called the oozooid), a colony of zooids develops by a weekly budding process. This process is comprised of four successive phases (A–D) [13], during which new zooids bud from the thoracic body wall of the oozooid and the subsequent zooids, a phenomenon called palleal budding [14] or blastogenesis [15–17]. Each blastogenic cycle ends in a massive apoptotic event (Phase IV—the takeover phase) in which parental zooids are resorbed concurrently with the maturation of buds into adult, functional zooids. A unique WBR event, in which buds develop at the bases of vascular ampullae, was observed in congener botryllid species under adverse environmental conditions [18,19], a regeneration phenomenon that had also been induced experimentally [10,11], providing a unique model system for WBR.

**Figure 1:**
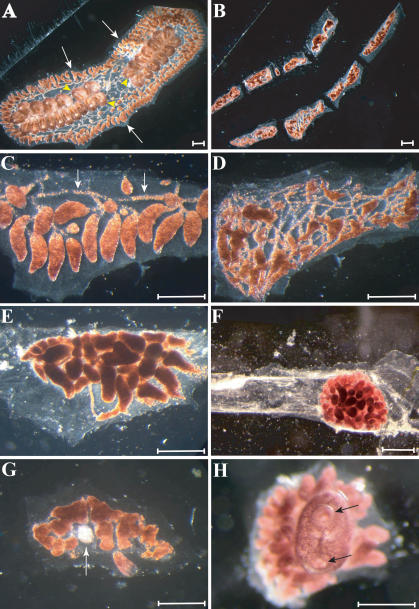
Morphological Events during WBR in B. leachi (A) A colony of B. leachi composed of two systems of genetically identical zooids (yellow arrowheads), each 2–3 mm long, embedded within the tunic. A network of blood vessels connects all zooids within a colony, from which pear-shaped vascular termini (ampullae) extend toward the colony margins (arrows). (B–H) Experimentally induced regeneration. The zooids and palleal buds in the center of the colony were cut out, leaving behind the marginal ampullae (B). Segments of several ampullae, 1–3 d after amputation, created a new blood vessel network (C) and (D), respectively (arrows). Isolated ampullae changed their orientations and shapes 6–7 d postseparation within the tunic matrix and coalesced into each other (E). Vasculature movements were more conspicuous when blood vessels were spaced out, creating a dense mass of vessels on one side of the fragment and a semitransparent gelatinous tunic matrix deprived of blood cells and blood vessels on the other side (F). By days 8–9, an opaque mass of blood vessels was formed, followed by the formation of an internal small transparent vesicle (G) (arrow). By days 10–14, a fully operating filter-feeding zooid equipped with functional atrial and peribranchial siphons, both facing upwards as in normal zooids, developed from the opaque mass of blood vessels (H) (arrows). The scale bar represents 1 mm.

Retinoic acid (RA) is a low molecular weight biologically active metabolite of Vitamin A, which plays diverse functional roles in a wide variety of tissues and organs [20–22]. It plays a conserved role in controlling positional information and cellular differentiation during embryonic development. RA action is mediated at the metabolism and signaling levels. RA-synthesizing enzymes, such as retinaldehyde dehydrogenase (Raldh), together with degrading enzymes (Cyp26) create the spatiotemporal distribution of RA during development and refine it [23]. Signaling is mediated through RA binding to two nuclear receptors, retinoic acid receptor (RAR) and retinoic X receptor, through which it acts as a ligand-dependent transcription factor [24,25], subsequently inducing gene transcription. RA has also been recorded as being involved in restricted regeneration of tissues and organs in adult vertebrates [26]. In urodele amphibians, administering RA into amputated limbs resulted in regeneration of extra limb tissue [27–29]. In adult rat lung, RA induces alveolar regeneration, and systemic RA treatment can reverse pathological features of experimental emphysema [30]. In adult rat spinal cord, RAR isoform RARβ2 is able to promote functional regeneration of sensory axons [31].

Despite the identification of RA receptors exclusively in chordates, and the consideration of RA signaling as a chordate innovation [32], remarkably little evidence exists connecting the activation of RA signaling to conserved chordate biological traits of regeneration. A single RA receptor [33] in lower chordates (such as urochordates and cephalochordates) offers an advantage when searching for direct RA-signaling functions. This is not the case in vertebrates where, due to redundancy in RAR genes, RAR localization fails to provide sufficient information on RA-signaling functions. Here, we endeavor to elucidate cellular processes and RA signaling during WBR in the urochordate B. leachi in order to reveal traits and evolutionary routes of regeneration similar to those recorded in its vertebrate counterparts [33].

## Results

### Morphological Changes in Amputated Ampullae

Experiments (*n* = 95) were conducted on small B. leachi fragments (each containing 1–92 isolated ampullae, cut from different colonies) that were well attached to the substrate. Whole body restoration was observed in 80 fragments, irrespective of the number of blood vessels, the blastogenic stage (A–D) of the colony on the day of operation, the original colony size (1–6 systems, 3–41 zooids), or the amputated vasculature location (peripheral, marginal, or from vessels located centrally) (Figure 1). Following surgery and isolation of vessel fragments (Figure 1B), we observed an immediate vasculature contraction and restriction of blood flow. The next day, vascular connections grew between ampullae within each fragment, creating a new circulatory system (Figure 1C and 1D, arrows). Then, blood flow was restored and blood cells were observed traveling (as in untreated colonies) in opposite directions, briefly in one direction and then back. At the same time, the lumen of some blood vessels became opaque and shrank progressively. During the first seven days, isolated ampullae were highly dynamic, changing their orientations and shapes within the tunic matrix, coalescing into each other, and moving within the tunic (Figure 1E and 1F). These directional movements and new vascular connections created a dense network of anastomosed vessels at selected locations within the tunic embedment. Vasculature movements were more conspicuous when blood vessels were spaced out, creating a dense mass of vessels on one side of the fragment and a semitransparent gelatinous tunic matrix deprived of blood vessels on the other side (Figure 1F).

Blood propulsion in the newly formed vascular network slowed down progressively. Concomitantly, an opaque mass of blood vessels was formed, followed by formation of an internal small transparent vesicle (Figure 1G, arrow) that grew and developed two openings in opposite corners (Figure 1H). By days 10–14, a fully operating filter-feeding zooid, equipped with functional atrial and peribranchial siphons (both facing upwards, as in normal zooids) (Figure 1H, arrows), developed from the opaque mass of blood vessels. The first generation of zooids was larger (1.18 mm^2^) than regular zooids (0.78 mm^2^) and exhibited a spherical shape, unlike the elongated pear-like shape of regular colony zooids. Irrespective of the number of blood vessels (1–92) presented in each fragment and of the number of buds (1–24) developing throughout the amputated fragment, only one zooid per fragment (*n* = 80) was fully formed. Yet, successful regeneration, indicated by the opening of siphons, varied between fragments, being faster (within 10 d) in fragments characterized by spacious blood circulation or fragments exhibiting early onset of a new vascular system. When a new vascular system failed to form properly between blood vessels, buds developed in between ampullae at vessel's joints. WBR was not documented in cases (*n* = 15) where fragments failed to separate completely from colonies, leaving a single zooid fragment or an intact blastogenic bud.

### Cellular Events

Regenerating vasculature fragments (*n* = 116) were sacrificed at sequential daily intervals and were processed for histological observations. Three distinctive morphological phases were identified at the cellular level. Regular cellular vasculature morphology was revealed by using control intact ampullae (*n* = 10).

#### Phase I: Dynamic reorganization in vessel architecture.

Following separation of blood vessel fragments from the colonies, circulating blood cells were found settling in distinct spots along the vasculature epithelium (Figure 2A, arrows), adhering to specific sites. Circulating haemocytes (see below) in isolated blood vessel fragments were stained by proliferating cell nuclear antigen (PCNA) (a proliferation marker), whereas weak or no PCNA staining was documented in haemocytes of intact blood vessels. No PCNA staining was documented in the surrounding peripheral epithelium. In isolated fragments, a dense network of cell connections formed gradually in the lumens of blood vessels. Within one day of separation, the epithelium detached from the surrounding tunic embedment, exposing a flattened and squamiform single-cell layer (Figure 2B, arrows). From day 2 onward, divisions within the blood vessel created dozens of small compartments (termed by us regeneration niches) varying in sizes and locations (see below).

**Figure 2:**
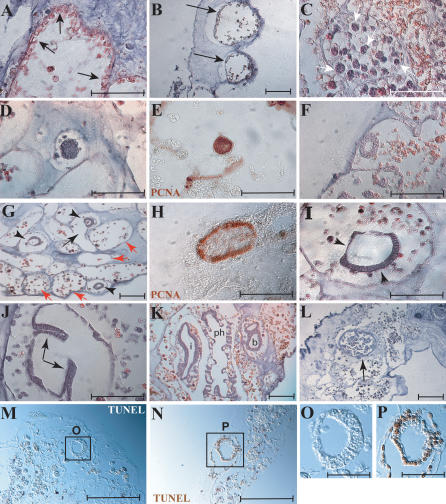
Histological Characteristics of WBR in B. leachi Regenerating vasculature fragments that were sacrificed at sequential daily intervals exhibit cellular events during the regeneration process. (A and B) Phase I events: Haemocytes are attached to vasculature epithelium (A) (arrows). Vascular detachment from the tunic (B) (arrows) enables subsequent relocation of blood vessels. (C–E) Phase II events: 2 d after dissection, aggregated haemocytes of various sizes and shapes were observed in the regeneration niches (C) (arrows). Extensive cell proliferation formed an opaque ball of cells (D) with exclusive PCNA staining (E). (F–L) Phase III events: 1 d later, continuous cell proliferation increased the size of cell aggregates and formed blastula-like structures of different shapes (F and G) (arrowheads). At this stage, multibuds developed simultaneously in various regenerating niches (G) (arrowheads and red arrows, respectively), although separation between compartments had not been completed (G) (arrow). PCNA specifically stained newly formed bud spheres and blastula-like structures inside the regeneration niches (H). As development proceeded, axial polarity was observed with the appearance of differential cell layers (I) (arrowheads). From day 5, two invaginations from both corners of the thick vesicle wall creating two elongated double-walled folds were observed (J) (arrows). Between 10–14 days postseparation, the regeneration process reached the final stages, whereby a fully functional adult zooid was developed, including the formation of palleal buds (K). Only one zooid per regenerating fragment reached this final stage, while the others degenerated (L) (arrow). (M–P) TUNEL analysis clearly shows staining in degenerating buds. Bud that was normally developed did not stain for TUNEL and exhibits a normal blastula-like structure with clear polarization: (M) and enlargement in (O). Bud that failed to develop went through a degenerating process and was stained for TUNEL: (N) and enlargement in (P). Note that it failed to develop a normal blastula structure, and cells started to fall apart. b, bud; i, intestine; ph, pharynx. Scale bar represents 100 μm. In (O) and (P) scale bars represent 30 μm.

#### Phase II: Initial stages of regeneration.

Multinucleated giant cells appeared at numerous locations throughout vasculature two days after dissection [10,11]. Afterwards, aggregates of haemocytes, of 2–32 cell clusters, developed inside regeneration niches. These haemocytes that initially were observed peripherally next to the surrounding epithelium, are characterized by high nucleo-cytoplasmic ratio, strong hematoxylin staining, round shape, and small cell size (4–5 μm). Haemocyte aggregates varied in size (8–20 μm in diameter) and form small spherically shaped cell masses (Figure 2C, arrows) (Table 1). In time, continuous cell divisions (see below) increased the numbers of cells in the aggregates, forming an opaque ball of cells (up to 35 μm in diameter) (Figure 2D and 2E) with heterogeneous cell numbers (up to 100). From day 2, the distinct PCNA staining was observed exclusively in the aggregated foci of haemocytes inside regeneration niches and in the cell balls (Figure 2F). The blood vessels' lumen was about 100–110 μm in diameter, and each large blood cell aggregate occupied about one-fifth of the vasculature volume. In fragments with many blood vessels (up to 92 in our experiments), a larger number of regeneration niches formed (Figure 2G, red arrows), culminating in the simultaneous development of many small buds (as many as 24 buds per experiment) (Figure 2C; Table 1). Newly formed buds occupied different compartments within the blood vessel, even if separation between compartments had not been complete (Figure 2G, black arrow).

**Table 1:**
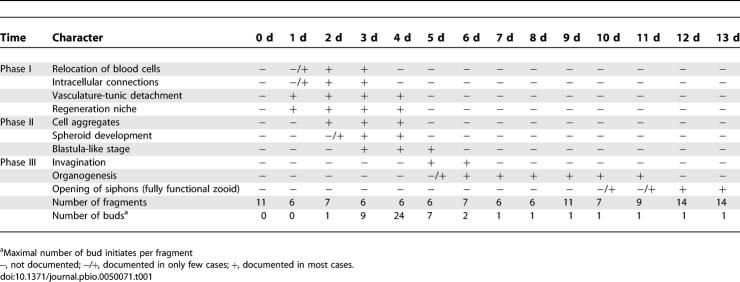
Characters for *Botrylloides* WBR

#### Phase III: The race for predominance.

From day 3 (Table 1), changes within aggregates resulted in the formation of a single-layer epithelial sphere, enclosing an internal cell-free transparent space (Figure 2F). These blastula-like structures varied in size (10–95 μm in diameter), cell numbers (20–200), and shape, from round spheres and bell shapes to elliptic structures (Figure 2F, 2G, arrowheads, 2H, 2I). In three-day-old isolated fragments, PCNA specifically stained newly formed bud spheres inside regeneration niches (Figure 2H). By day 4 (Table 1), structural changes created blastula-like forms that revealed axial polarity on the cellular level. A thin single squamiform layer characterized the distal (from the vessel epithelium) side of the spheroid. Numerous cell layers were observed at the opposite pole facing the epithelium (Figure 2I).

Continuous cell proliferation led to bud enlargement, which was significant because small buds failed to develop further (Table 1). As of day 5, development proceeded by two invaginations from both corners of the thick vesicle wall, creating two elongated double-walled folds (Figure 2J, arrows). These folds gradually extended inward until the vesicle divided into a median and two lateral chambers, from which the prospective central pharyngeal chamber and a pair of lateral atrial chambers developed. Subsequently, both double-walled folds thickened, forming the sites for the future gill slits. In all cases but one, only a single bud per fragment reached this stage of organogenesis. Thereafter, internal organs continued to grow into a functional, sexually reproductive, filter-feeding adult zooid (Figure 2K). In all cases observed, the fastest developing buds reached final stages of organogenesis, whereas the development of other buds halted (Figure 2L, arrow); then they degenerated through an apoptotic process (Figure 2M–2P) and were subsequently absorbed.

### Cloning and Expression Pattern of the RAR Homologue during WBR

A homologue of RAR (Bl-RAR) was cloned from the cDNA of regenerating blood vessels. An 808-bp fragment was sequenced, revealing 87% identity to RAR of the budding tunicate Polyandrocarpa misakiensis. A domain search revealed a conserved 157-amino acid fragment, corresponding to the ligand-binding domain of hormone receptors present in all RARs [34], (Figure 3A, red line). Furthermore, Bl-RAR is phylogenetically clustered with other urochordate RAR family members in a general glade of other vertebrate RARs and is significantly distinguished from other RA receptor families (Figure 3B).

**Figure 3:**
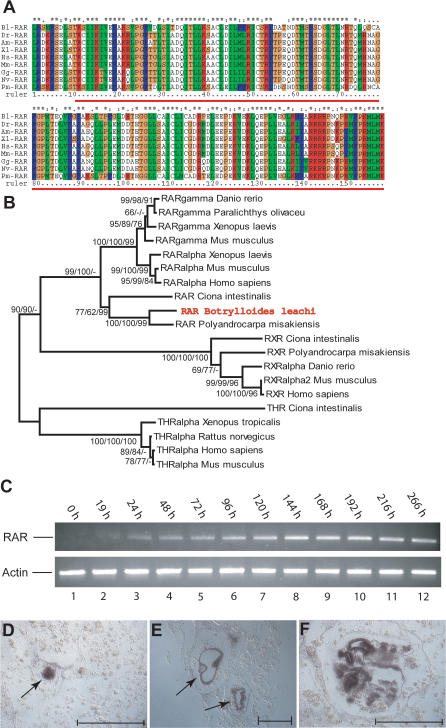
Cloning and Expression Pattern of the RA Receptor Homologue during WBR (A) A fragment of 157 amino acid exhibiting high homology to the ligand-binding domain (marked by a red line) of hormone receptors present in all RARs. (B) An evolutionary tree of different RARs, retinoic X receptors, and thyroid hormone receptor members of this family of RA receptors in different urochordates and vertebrates. Molecular evolutionary analysis was conducted using the MEGA 2.1 program using the tree-making method Minimum evolution. The numbers along the outer side of each branch represent the bootstrap values in percentage (1,000 reps) of three different tree-making methods: the selected tree, Minimum evolution/ Neighbor-joining/ Maximum parsimony. The sign “-“ marks bootstrap values that are less than 50% or a complete different outcome of the specific branch compared to the same branch in the selected tree. (C) RT-PCR analysis was performed on regenerating ampullae at different developmental stages. Intact blood vessels (the controls) did not express the RAR transcript (lane 1) while as early as 19 h postseparation, the transcripts were detectable and showed a gradual increase in their levels along the entire process (lanes 2–12). Actin levels remained equal representing the viability of the regenerating ampullae and serve as a positive control to normalize RAR levels. (D–F) Whole-mount RNA in situ hybridization was employed on paraffin sections of regenerating ampullae at different developmental stages. Specific staining pattern was visible from day 2 (D) (arrow) in aggregates of blood cells in the regenerating niches. In cases where several regeneration niches were established and buds developed simultaneously within the ampullae, RAR expression pattern was localized specifically to all buds (E) (arrows). Later on, RAR continued to specifically stain regenerating buds through the subsequent developmental stages of spheres, invaginations and organogenesis (F). Scale bar represents 100 μm. Bl, *B. leachi;* Brl, *Branchiostoma lanceolatum;* Dr, *Danio rerio;* Gg, *Gallus gallus;* Hs, *Homo sapiens;* Mm, *Mus musculus;* Nv*, Notophthalmus viridescens;* Pm, *P. misakiensis;* Xl, *Xenopus laevis.*

To gain insight into the temporal expression pattern of RAR during regeneration, total RNA was extracted from fragments of regenerating blood vessels at different developmental stages, and RT-PCR was performed using specific primers. Intact blood vessels (controls) did not express the RAR transcript (Figure 3C, lane 1). Similar outcomes were obtained when PCR product from control ampullae was amplified 40 cycles, revealing no traces of the transcript. In contrast, RAR transcript was detected in cDNA of regenerating ampullae as early as 19 h after isolation from the colony (Figure 3C, lane 2). A PCR transcript was expressed continuously in PCR products throughout subsequent regeneration stages for up to 11 d (Figure 3C, lanes 3–12).

The spatial expression of RAR was assessed during WBR. Blood vessels were separated from B. leachi colonies and left to regenerate in 1-l plastic tanks containing seawater. Intact and regenerating vasculature fragments were fixated at temporal intervals, and in situ hybridization was employed on 5-μm thick paraffin sections. Intact blood vessels showed no detectable hybridization with the probe (unpublished data), while regenerating blood vessels showed a specific staining pattern that was visible from day 2 (Figure 3D, arrow). The early expression pattern of the RAR probe specifically demarcated regeneration sites in aggregates of haemocytes at various niches within the blood vessels. In cases where several regeneration niches were established and buds developed simultaneously within the ampullae, RAR expression pattern was localized specifically in all buds (Figure 3E, arrows). Later on, RAR continued to stain, specifically, regenerating buds through the subsequent developmental stages of spheres, invaginations, and organogenesis (Figure 3F). No detectable staining had ever been observed in blood vessel epithelium or circulating blood cells that did not participate actively in regeneration. Specific sense probes were used as controls revealing no detectable staining pattern (unpublished data)**.**

### Cloning and Expression Pattern of a B. leachi Raldh Orthologue during WBR

In order to identify cellular sources of RA synthesis and metabolism, a 337-bp fragment was amplified and subsequently cloned from cDNA of regenerating blood vessels. The deduced amino acid was most similar to Aldehyde dehydrogenase (Aldedh) from the budding ascidian P. misakiensis (*E*-value = 4e−55) and revealed high sequence similarity to Aldedh2 family members from chick and mouse (66% identity, *E*-value = 2e−42) (Figure 4). A domain search revealed an Aldedh domain (Figure 4A, red line) conserved in all Aldedh family members [35].

**Figure 4:**
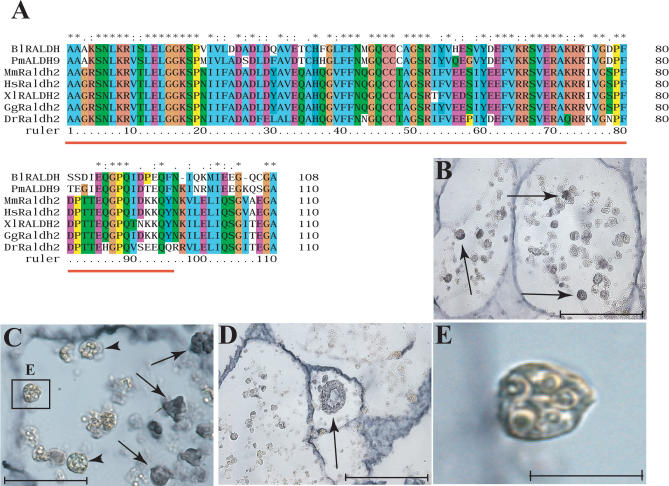
Cloning and Expression Pattern of the Raldh Homologue in Intact Colonies and Regeneration Ampullae (A) A fragment of 110 amino acid exhibiting high homology to the Aldedh domain of the budding ascidian P. misakiensis (Pm) and high sequence similarity to Aldedh2 family members from mouse (Mm), Human (Hs), Xenopus (Xl), chick (Gg), and zebrafish (Dr). A domain search revealed an Aldedh domain conserved in all Aldedh family members (98 amino acid marked by a red line). (B–E) Expression pattern of Bl-Raldh in intact colony and regenerating ampullae. Bl-Raldh is expressed in naïve ampullae, exclusively in a population of circulating macrophage cells (B) (arrows), scattered throughout vasculature. In regenerating ampullae, Bl-Raldh is observed in macrophage cells (C) (arrows) adjacent to aggregates of small cells creating morula-like structures (C) (arrowheads, rectangle), enlargement in (E). In later stages, starting from the blastula-like stage, Bl-Raldh expression appears in developing buds (D) (arrow). Note the staining along the ampullae margins represents nonspecific staining of the tunic matrix. Scale bars in (B) and (D) represent 100 μm. Scale bars in (C) and (E) represent 40 μm and 10 μm, respectively.

B. leachi Raldh (Bl-Raldh) expression was analyzed by in situ hybridization on sections of naïve and regenerating blood vessels and revealed a distinct expression pattern during WBR. In naïve ampullae, Bl-Raldh is expressed exclusively in a population of circulating macrophage cells scattered throughout the vasculature (Figure 4B, arrows). Following amputation, RA synthesis was documented in macrophage cells (Figure 4C, arrows) in regeneration areas adjacent to nonstained, small cell morula-like aggregates (Figure 4C, arrowheads, rectangle and Figure 4E enlargement). During the regeneration process, Bl-Raldh was not expressed in lymphocyte-like cells, morula-like cells and structures, other common blood cell types, or in the surrounding vessel epithelium. Concomitantly to its strong expression levels in macrophage cells, starting from the blastula-like stage, Bl-Raldh displayed expression patterns overlapping RAR expression throughout bud development (Figure 4D, arrow). A similar correlate expression pattern of RAR and Raldh was observed throughout colony astogeny (unpublished data). Specific sense probes were used as controls revealing no detectable staining pattern (unpublished data)**.** Therefore, the sequence similarity between Bl-Raldh and other vertebrate Raldh molecules combined with the specific parallel expression patterns of Bl-Raldh and Bl-RAR during WBR and colony astogeny classifies this gene product as a Raldh family member. Following the documented complexity of the Aldedh gene family in urochordates [36], we cannot exclude the presence and/or interplay of additional Raldh genes during WBR.

### Specific RA Synthesis Inhibitors 4-Diethylaminobenzaldehyde and Geranial, 3,7-Dimethyl-2,6-Octadienal Inhibit Bud Regeneration

To assess the functional roles of RA synthesis during WBR, peripheral ampullae were dissected from colonies and left to regenerate in 1-l plastic tanks containing the RA synthesis inhibitor 4-Diethylaminobenzaldehyde (DEAB). For control purposes, peripheral ampullae were separated from B. leachi colonies and left to regenerate in 1-l tanks containing DMSO alone. We conducted two sets of regeneration groups employing different DEAB concentrations (10 μM and 100 μM) (Table 2). Both experimental groups produced similar morphological outcomes. Characteristics of early-stage regeneration were similar between experimental cases (*n* = 22) and controls (*n* = 12) and also showed similarity to the previously characterized cases (*n* = 95). DEAB-treated regenerating ampullae shrank. While blood vessels in these fragments started directional movements inside the tunic matrix (Table 2), complete ampullar aggregations in the form of opaque masses of ampullae were never documented (*n* = 22). Instead, vessel coalescence had gradually slowed down and stopped completely after day 2, leaving blood vessels in an intermediate state (Figure 5A; compare to Figure 1F). In control fragments, as in the former studied cases, zooids appeared 10–14 days postsurgery. In contrast, no single zooid had ever developed in any of the DEAB-treated fragments (Table 2), even after a follow-up observational protocol of 30 d (*n* = 11) in which all DEAB-treated blood vessel fragments eventually degenerated. Similar results were obtained with the RA synthesis inhibitor Geranial, 3,7-Dimethyl-2,6-Octadienal (Citral). Two experimental groups were conducted using different concentrations of Citral, showing a dose-dependent phenotype, ranging from complete attenuation of vessel movements (at 60 μM) to complete aggregations (at 20 μM) (Figure 5B and 5C, respectively; Table 2). Similar to DEAB treatments, no regeneration was documented even after a follow-up observational protocol of 30 d (*n* = 23) (Table 2).

**Table 2:**
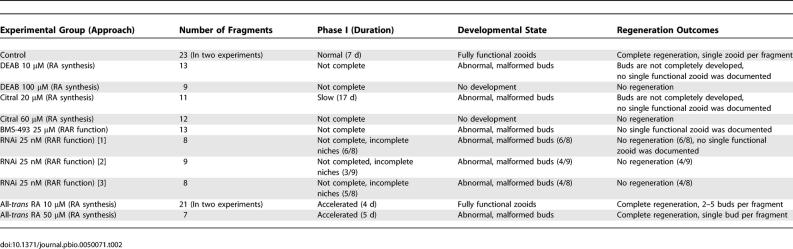
Outcomes of Gain-and-Loss of Function Experiments for RA Signaling during WBR

**Figure 5:**
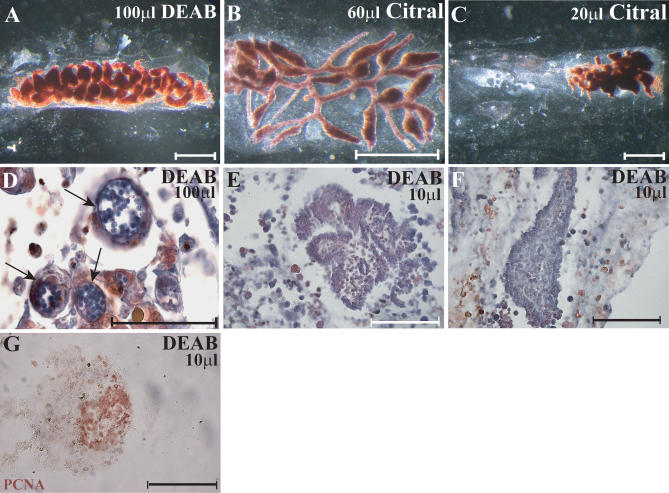
Specific RA Inhibitors, DEAB and Citral, Inhibit Bud Regeneration Early stages of bud regeneration were blocked by different concentrations of RA inhibitors. With the administration of 100 μM of DEAB, vessel coalescence gradually slowed down and stopped completely after day 2 (A). Similar results were obtained with 60 μM of Citral (B), while with 20 μM of the same inhibitor ampullae changed orientation and migrated within the tunic but never regenerated a functional zooid (C). At the 100 μM DEAB concentration, buds had not developed, and multinucleated giant cells appeared, scattered throughout the vessel lumen (D, arrows). At the 10 μM DEAB concentration, morphologically abnormal buds were formed, which failed to develop organ structures, and retained a simple epithelial morphology (E). In severe cases, masses of undifferentiated aggregated cells occupied the interior of the vessel lumen (F). All malformed buds subsequently degenerated. PCNA immunostaining revealed distinctive proliferations at aggregated masses while no staining was detected in blood vessels (G). Scale bar in (A–C) represents 1 mm. Scale bar in (D–G) represents 100 μm.

We sacrificed 10-μM and 100-μM DEAB-treated and control fragments for histological observations at temporal regeneration intervals. Controls showed the regular regeneration morphology as specified earlier. In DEAB-treated fragments, internal lumen was compartmentalized and taken over by partially formed regeneration niches. Yet, at both DEAB concentrations, there was an unusual increase in cell density inside the vessels' lumen, with more connections between cells (unpublished data). At 100-μM DEAB concentration (*n* = 9), buds did not develop and multinucleated giant cells appeared, scattered throughout the vessel lumen (Figure 5D, arrows). At 10-μM DEAB concentration (*n* = 13), morphologically abnormal buds formed that failed to develop organ structures and retained a simple epithelial morphology (Figure 5E). In severe cases, masses of undifferentiated aggregated cells occupied the interior of the vessel lumen (Figure 5F). All malformed buds subsequently degenerated. PCNA immunostaining revealed distinctive proliferation at aggregated masses, while no staining was detected in blood vessels (Figure 5G).

### Disruption of RAR Function during Regeneration Causes Bud Malformations Similar to RA Synthetic Inhibition

We disrupted RA receptor function by way of RNA interference (RNAi) as well as the RAR pan-antagonist BMS-493 separately. Ampullae, surgically separated from B. leachi colonies, were soaked for 6 d in seawater containing control small interfering RNAs (siRNAs) and siRNAs generated against Bl-RAR, as previously shown [37]. Outcomes were morphologically monitored daily followed by histological analyses. Knockdown of Bl-RAR expression was verified in intact colonies after 6 d of incubation by comparing the abundance of RAR to actin transcripts (Figure 6A). As clearly seen in siRNAs-treated colonies, RAR expression was nearly eliminated (Figure 6A, lane 2) while no significant change was observed in the RNAi control experiment (Figure 6A, lane 1) compared to naïve colonies (Figure 6A, lane 3). Differences between control RNAi and RNAi experiment fragments were visible as of day 2, when blood vessels moved actively in control fragments, compared to arrest movement in siRNAs-treated fragments (unpublished data). After 6 d, control experiments using control siRNAs completed vasculature movements, forming opaque vessel masses as previously specified (Figure 6B). In contrast, no single opaque vessel mass appeared in RNAi affected experiments (*n* = 14). Instead, blood vessels remained apart, situated haphazardly within the tunic matrix as in the premature stages (Figure 6C). In several experiments, vessel movement halted in an intermediate state, similarly to the chemically inhibited experiments (Figure 6D). A similar phenotypic morphology was observed with the RAR pan-antagonist BMS-493 (Figure 6E; Table 2). Both controls, RNAi and BMS-493-treated experiments, were histologically examined in hematoxylin-eosin stained sections (10 μm). The regenerating buds in RNAi treatments were malformed (Figure 6F, arrow; Table 2), and the regeneration niches were mostly taken over by disorganized masses of undifferentiated cells. In mild cases, buds reached progressive developmental stages but failed to invaginate properly (Figure 6G, arrow). A similar histological phenotype is observed in BMS-493-treated fragments exhibiting malformed epithelial spheres (Figure 6H, arrows; Table 2).

**Figure 6:**
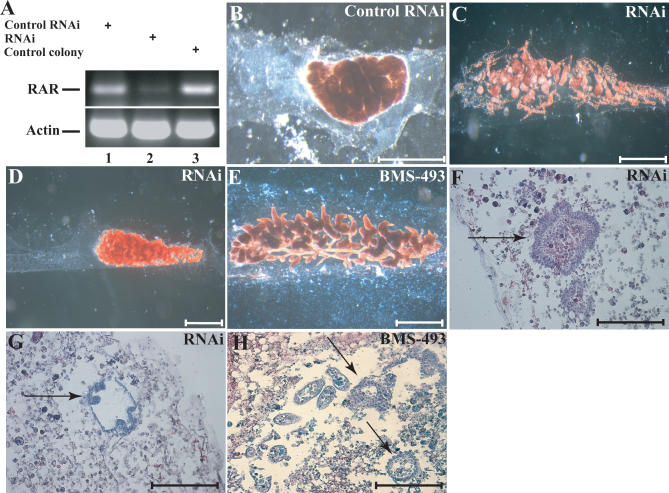
Disruption of RAR Function during Regeneration Causes Bud Malformations In order to verify the efficiency and ability of RAR siRNAs to interact and decrease RAR RNA levels, we added RAR siRNAs and control siRNAs to B. leachi colonies and compared RAR RNA levels to untreated colonies. Control siRNAs and untreated colonies exhibit approximately the same transcripts levels (A) (lanes 1 and 3), while RAR siRNAs show a significant reduction in RAR RNA levels (A) (lane 2). Actin transcript levels remain unchanged, indicating the viability of the tissue. After 6 d, control experiments using the control siRNAs completed vasculature movements, forming opaque vessel masses as specified before (B) (compare to Figure 1F). In contrast, in RNAi-affected experiments, blood vessels remained apart, situated haphazardly within the tunic matrix (C) (compare to Figure 5A). In several experiments, vessel movement halted in an intermediate state (D). A similar phenotypic morphology was observed with the RAR pan-antagonist BMS-493 (E). Histological sections reveal malformation of regenerating buds in RNAi treatments (F, arrow, compared to Figure 5E and 5F). In mild cases, buds reached progressive developmental stages but failed to invaginate properly (G, arrow). A similar histological phenotype is observed in BMS-493-treated fragments exhibiting malformed epithelial spheres (H, arrows). Scale bar in (B–E) represents 1 mm; scale bar in (F–H) represents 100 μm.

### All-*trans* RA Leads to Accelerated Regeneration and Multibud Formation

Next, we examined the gain-of-function effects of RA on WBR by the systemic administration of 10 μM or 50 μM all-*trans* RA to regenerating blood vessel fragments (*n* = 28) (Table 2). Control blood vessel fragments (*n* = 11) were immersed in seawater containing DMSO alone. On day 6, control fragments were still in the midregeneration stage, characterized by movements within the tunic matrix. However, in RA-treated fragments, vessels had already completed orientation within the tunic matrix (day 1), completed vasculature aggregations (days 1–4), and developed into opaque vessel masses. Furthermore, at the early day-6 stage, 11 of the RA-treated fragments (39%) had already developed fully functional adult zooids from the vessel masses with active atrial and peribranchial siphons.

On day 6, all RA-treated fragments were sacrificed for histological examinations. Results showed further accelerated regeneration throughout the entire vasculature. Haemocytes aggregated systemically along blood vessels and not only near the vessel epithelium. Buds were present in many blood vessels and revealed extremely high variation in developmental states, from cell aggregates to well-developed zooids. The regenerating zooids manifested fully differentiated organ systems (Figure 7; Table 1), including stigmata with cilia (Figure 7A, arrows), a completed digestive system, and a pair of contracting siphons carrying normally developed tentacles. Different from normal blastogenic buds, blood cells colonized extensively these regenerating buds between atrial folds and throughout internal cavities (Figure 7B, arrows). The consecutive generation of palleal buds, at progressive developmental stages, had already started to grow from both sides of the regenerating zooids. In one case, the regenerating bud progressed to the stage where secondary buds formed on the primary palleal buds (Figure 7C, arrow and arrowhead, respectively). In marked contrast to control regenerating fragments where only a single bud developed, numerous buds at different regeneration niches had simultaneously reached the final stages of organogenesis (2–5 functional zooids) (Figure 7D, arrows), exhibiting fully differentiated organ systems that reproduced asexually through palleal budding. At high concentrations of RA (50 μM), buds developed abnormally with an enlarged digestive tract, pyloric gland, and swelling of the atrial folds (unpublished data).

**Figure 7:**
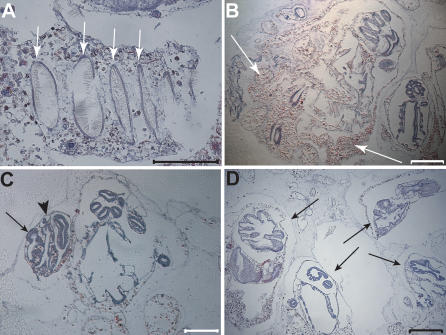
All-*trans* RA Leads to Accelerated Regeneration and Multibud Formation RA-treated regenerating fragments that were sectioned at different final stages of the regeneration process show a remarkable accelerated regeneration. By days 6–7 postseparation, most of the regenerating buds reached these final stages (compared to 10–14 days in control experiments), exhibiting complete organogenesis. The regenerating zooids manifested fully differentiated organ systems such as stigmata with cilia (A, arrows). Different from normal blastogenic buds or control experiments, blood cells extensively colonized these regenerating buds between atrial folds and throughout internal cavities (B, arrows). The acceleration of the process was also exemplified in the subsequent blastogenesis. At day 7, the regenerating bud progressed to the stage where secondary buds were formed on the primary palleal buds (C, arrow and arrowhead, respectively). In marked contrast to control regenerating fragments, where only a single bud was developed, numerous buds at different regeneration niches simultaneously reached the final stages of organogenesis (2–5 functional zooids) (D, arrows, compare to Figure 2G). Scale bar represents 100 μm.

## Discussion

Whereas most animals share the ability to repair damaged tissue, the phenomenon of massive body restoration is a rare event in highly complex animals [5]. Here, we studied the unique whole body regeneration phenomenon in a colonial urochordate, revealing the capability of rebuilding the entire zooid body from minute vasculature fragments. We defined three consecutive developmental phases, shedding light on a unique regeneration process that differs from known regeneration model systems in several fundamental criteria (Table 3). In other epimorphic regenerations, restoration starts with the formation of a transient structure termed blastema [38]. However, in the *Botrylloides* WBR, instead of blastema, regeneration niches are structured de novo inside vasculature. Furthermore, while a single restoration center is induced in all other model systems studied [5], a “multifocal regeneration niche” system is generated in the *Botrylloides* WBR. A possible explanation for this regeneration strategy in *Botrylloides* is the presence of a systemic inductive regeneration cue. Regeneration in Animalia is regularly induced by inductive signals [39,40]. In model systems, such as regeneration of limb in salamander or fin in zebrafish [41,42] it is regulated by locally induced signals. We suggest that in *B. leachi,* WBR, in contrast to other cases, signals might be propelled throughout circulation (cellular and humoral elements), leading to many regeneration foci in any single regeneration entity. The *Botrylloides* system is also the first documented case, to our knowledge, of WBR originating from circulating blood cells (Table 3) and reveals the capability of restoring not only the soma, but also the germ line [10,11].

**Table 3:**
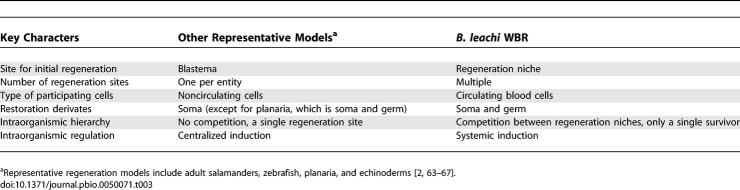
Depiction of Unique Regeneration Characteristics in the B. leachi WBR Model System

As previously reported [10,11,19], *Botrylloides* WBR initiates through aggregates of haemocytes within newly formed vasculature niches; each housing up to 100 small round cells. Any haphazardly isolated peripheral ampulla (out of hundreds of ampullae fringing in a single colony), containing only 100–300 circulating cells [10,11], is capable of successful WBR. What is the cellular source of the regenerating bud? Their small cell size, cellular morphology, and strong ubiquitous hematoxylin staining suggest primitive stem cell origin [19]; however, direct proof should be addressed in further controlled studies.

In *Botrylloides* WBR, as in embryonic development [43], the transformation of the initial state of aggregated cells into complete animal structures reveals a fundamental attribute of animal development. Both processes have very different starting points (a fertilized egg versus pluripotent blood cells); yet both converge at a common endpoint of adult morphology through the establishment of body axes, self-assembly, and differentiation of tissues and organs [44,45]. We can draw close morphological similarities between the initial developmental stages in *Botrylloides* WBR (cell aggregates, development of blastula-like structures, and invaginations of epithelial layers) and fundamental embryonic developmental stages (morula, blastula, and gastrula). A similar resemblance transpires when comparing *Hydra* WBR from dissociated cells [46] to embryonic development, implying a basic evolutionary root underlying whole body restoration events [47,48].

The conserved signaling pathways governing the development of tissues and organs are one of the major objects in developmental biology. Redeployment of these embryonic signaling pathways at different biological contexts enables quicker responses by multicellular organisms to adverse environmental conditions, such as RA signaling in the regeneration of specific tissues and organs in various vertebrate model systems [26,49,50]. A functional example of redeployment of a developmental mechanism comes from the mammalian central nervous system. Embryonic central nervous system undergoes regeneration through RARβ2-stimulated RA signaling, unlike adult central nervous system, which does not express RARβ2. Redeployment of RA signaling through overexpression of RARβ2 indeed promotes functional regeneration of adult central nervous system [31].

It had already been shown that RAR is expressed in mesenchymal cells of developing buds in the colonial tunicate *P. misakiensis,* where it regulates developmental aspects in normal budding processes, including the induction of a secondary axis in developing buds [51–53]. Following the evaluation of the biological redeployment strategy, this study reveals, for the first time to our knowledge, the important roles of RA in botryllid ascidian WBR. We show that RAR is expressed during WBR, specifically in haemocytes within the vasculature. In contrast to tissue- and organ-specific expression patterns of RA receptors in vertebrates, RAR expression in botryllid regeneration does not follow developmental processes expressed in normal blastogenesis of tissue and organ structuring, but is expressed ubiquitously throughout the entire body. This observation suggests that in addition to its role in early stages of regeneration, RAR expression also represents other RA activities, such as maintenance or other yet unknown proceedings. We further show that the entire body of developing zooids is susceptible to RA signaling. The use of both DEAB, Citral, BMS-493, and RNAi-mediated knockdown of Bl-RAR to disrupt RA synthesis and RA receptor function, collectively result in WBR arrest and bud malformation. The administration of all-*trans* RA into fragments of blood vessels results in accelerated regeneration and the unusual development of multibuds, leading to restored colonies with multiple functional zooids. This observation could indicate that in regular *Botrylloides* WBR, the systemic developmental inhibition of all buds but one, could be achieved by controlling the levels of RA transcriptional cascade. We found, during early stages of WBR, that highly Aldedh-positive macrophages form foci of expression that correspond to foci of multiple initiations of regeneration, strengthening the notion that RA is required for the early stages of this process [54,55].

Regeneration as a central discipline in biology holds great promise not only for the understanding of species-specific developmental issues, but also for deduction of its evolutionary roots and for medical applications [56]. In the past few years, ascidians have become model organisms for the study of a wide variety of biological phenomena, including developmental processes, embryogenesis, stem cell biology, and immunology [57–60]. This group of organisms demonstrates basic mechanisms of biological phenomena, similar to those observed in vertebrates. In light of the unique regenerative power of botryllid ascidians, WBR in B. leachi may serve as a model system for studying the evolutionary roots of organ regeneration, lost in most vertebrate taxa.

## Materials and Methods

### Animal husbandry and colony dissection.

Colonies of B. leachi were collected from underneath stones in shallow waters along the Mediterranean coast of Israel. The colonies, with thin layers of stony material attached to them, were carefully pealed off from the substratum using industrial razor blades and individually tied with fine threads onto 5 × 7.5-cm glass slides. Colonies were cultured on slides placed in 17-l tanks of standing seawater system [33]. Within several days of culture, ampullar contractions and expansions resulted in complete or partial sliding of colonies from their natural calcareous substrate onto the glass slides. Colonies were cleaned weekly by carefully removing debris (empty substrates and other settled organisms) from the glass slides with industrial razor blades and fine brushes.

Isolation of marginal ampullae and blood vessel fragments was performed under a dissecting microscope using an industrial razor blade and a fine tungsten needle. Next, the dissected colonies were removed from the glass slides onto other slides, and the colonial fragments were cut further into smaller fragments using a fine tungsten needle. Blood vessel fragments were left to regenerate in 17-l tanks and were monitored daily by observing slides under a dissecting microscope. Whole fragment pictures were taken with a Supercam camera (Applitec, http://www.applitec.co.il).

### Histology and immunohistochemistry.

Immunohistochemistry and general histology were performed as described by Lapidot [61]. Dissected blood vessels were fixed in Bouin's solution for 1 h, dehydrated in 70% ethanol, and embedded in paraffin wax. For general histological observations, we used the regular hematoxylin-eosin staining protocol. For immunohistochemistry, serial 5-μm thick sections were prepared, attached to SuperFrost Plus microscope slides (Menzel-Glaser, http://www.menzel.de), dewaxed, and antigen retrieval was performed by microwaving (480 W) the sections for 30 min in 10-mM citrate buffer (600 ml, [pH 6], 30 min). After a 5-min cooling period, up to 1 l in volume distilled water was added, and the slides were incubated for additional 10 min at room temperature, followed by several washes with TBS. Endogenous peroxidase activity in the sections was blocked by incubation in Dako EnVision^+^ System Peroxidase block (AEC, catalogue number K4005, http://www.dako.com,) for 6 min, and the slides were washed with water. Nonspecific binding sites were blocked by incubation in 1% BSA (Sigma, http://www.sigmaaldrich.com) in 50 mM TBS−0.1% Tween 20−0.01% Triton × 100 for 1 h at room temperature. The slides were washed with TBS for 10 min, stained with a mouse anti-PCNA monoclonal antibody (Dakocytomation, 1:100 in TBS, 100 μl per slide) for 1 h at room temperature, and then incubated for 12 h at 4 °C. After washing with TBS for 5 min × 3, peroxidase-labeled polymer-conjugated goat anti-mouse immunoglobulin (Dako EnVision^+^) was added; the sections were incubated for 1 h and washed with TBS for 5 min × 3. Then, 2–3 drops of AEC substrate, chromogen (3-amino-9-ethylcarbazole hydroperoxide, Dako, EnVision^+^) was added for 20–60 min (upon judgment), and the reaction was stopped by rinsing with distilled water. Control and experimental sections were mounted with Hydromount (National Diagnostics, http://www.nationaldiagnostics.com), covered with 24 × 50-mm cover glasses (Medite, http://www.medite.ch), observed with the Leica DMIRE2 inverted microscope, and photographed with a Leica FX300 camera (http://www.leica.com).

### Isolation of the RAR, Raldh, and cytoplasmic actin homologues from B. leachi and analysis of endogenous transcripts by RT-PCR.

Total RNA was isolated from fragments of regenerating blood vessels with RNeasy Mini or Midi kits (Qiagen, http://www.qiagen.com).

A cDNA fragment of 808 bp was amplified using degenerate oligonucleotide primers (forward: GGVTGYAAGGGITTCTT; reverse: TTCATVAKCATYTTIGGGAA) directed against two conserved regions of the ligand-binding domain of hormone receptors present in all RARs. Amplification was obtained using Bl-RAR sequence specific primers (forward: TCGACGCTTTCGGGCATAC, reverse: AAGACGGCAAAGCGGGAGAG). A B. leachi Raldh cDNA fragment of 337 bp was cloned from cDNA of regenerating blood vessels and amplified using sequence specific primers (forward: AGAATTTCCTTGGAGCTTGG; reverse: ACCCTGTTCAATGTCGCTG). A B. leachi cytoplasmic actin cDNA fragment of 338 bp was cloned from cDNA of B. leachi colonies and amplified using the following primers (forward: GAAATCGTGCGTGACATCAAAG; reverse: GCGGTGATTCCCTTCTGCATAC). BLAST results confirmed it as a cytoplasmic actin. PCR products of B. leachi Actin, RAR, and Raldh were cloned using pGEM-T easy vector (Promega, http://www.promega.com and Qiagen) PCR cloning kit, respectively. Sequences related to Bl-RAR and Bl-Raldh were identified with BLAST, and ClustalW was used to obtain the multiple alignment of both sequences. All sequences of RAR- and Raldh-related proteins used for the multiple alignments were obtained from the EMBL Nucleotide Sequence Database (http://www.ebi.ac.uk/embl/).

RT-PCR analysis was performed by extracting total RNA from regenerating blood vessels at temporal stages using RNeasy Mini or Midi kits (Qiagen) as a template. After cDNA production by reverse transcriptase, PCR reactions were performed (40 cycles at 94 °C for 30 s, 30 s at 50 °C, and 60 s at 72 °C) using specific primers corresponding to Bl-RAR and Bl-cytoactin.

### In situ hybridization.

B. leachi colonies, colonial fragments containing blood vessels, and regenerating fragments were fixed overnight in 4% paraformaldehyde, dehydrated in 70% methanol, embedded in paraffin, and cut into 5-μm sections. Both Bl-RAR and Bl-Raldh clones were used to obtain sense and antisense DIG-labeled RNA probes that were synthesized using the DIG RNA labeling kit (SP6/T7, Roche Molecular Biochemicals, http://www.roche.com). Hybridization of probes to tissue sections was performed according to Breitschopf [62] for paraffin-embedded tissue. DIG-labeled RNAs on samples were revealed using anti-DIG antibody (Roche). Samples were observed with the Leica DMIRE2 inverted microscope and photographed with a Leica FX300 camera.

### RA, Citral, DEAB, and BMS-493 administration.

Stock solutions were made in the dark. All experiments were carried out in aluminum foil covered plastic tanks. Solutions were changed every other day. All-*trans* RA was purchased from Sigma, and a 0.1-mM stock solution was made by adding DMSO. Stock solutions were frozen in −20 °C as aliquots of 100 μl. Immediately prior to experiment, an aliquot of stock solution was thawed and further diluted when added in different concentrations to the experimental 2-l seawater tanks. Citral (*cis* + *trans,* Fluka [Sigma]) 60-mM stock solution was prepared in 100% ethanol and was further diluted to 20 μM and 60 μM by administrating into 1-l seawater tanks. We prepared DEAB (Fluka) stock solution by dissolving 0.177 g DEAB in 1 ml DMSO within an aluminum covered Ependorf vial. The stock solution was further diluted to 10 μM and 100 μM by adding to 1-l seawater tanks. We prepared 25-μM BMS-493 by diluting 20 μl of 25 mM stock solution in 20 ml seawater.

Glass slides with B. leachi fragments of different regeneration phases were placed in the water tanks, left to regenerate, and examined daily under a dissecting microscope. Whole fragments' pictures were taken with a Supercam camera (Applitec).

### RNA knockdown experiments.

Three different siRNAs to Bl-RAR were generated using the Silencer siRNA Construction kit (Ambion, http://www.ambion.com). The specific primers used were as follows: As1 5′-AAATGGTATGCCCGAAAGCGTCCTGTCTC-3′ and S1 5′-AAACGCTTTCGGGCATACCATCCTGTCTC-3′; As2 5′-AAACTACAAGAACCTCTCGTCCCTGTCTC-3′ and S2 5′-AAGACGAGAGGTTCTTGTAGTCCTGTCTC-3′; As3 5′-AACGCAACTTCAAAGTTGCGCCCTGTCTC-3′ and S3 5′-AAGCGCAACTTTGAAGTTGCGCCTGTCTC-3′. Three additional sets of primers and an unrelated control siRNA [37] were generated by Ambion according to the Bl-RAR sequence provided. siRNA was delivered by the submersion of animal colonies for 2 d, followed by separating the blood vessels from the colonies and immersing the regenerating blood vessels in seawater containing 25 nM siRNA for an additional 6 d, during which the medium was changed every other day. Control siRNAs and siRNA-treated regenerating blood vessels were monitored daily and collected for further analyses.

### Klenow fragEL apoptosis assay.

Apoptotic nuclei were stained using a Klenow fragEL DNA fragmentation detection kit (TUNEL) (QIA21, Calbiochem, http://www.emdbiosciences.com/CBC) according to the manufacturer's protocol of paraffin-embedded tissue. Negative controls were generated by substituting the Klenow enzyme in the labeling reaction mix with dH_2_O.

## Supporting Information

### Accession Numbers

The EMBL Nucleotide Sequence Database (http://www.ebi.ac.uk/embl) accession numbers cDNA fragments discussed in this paper are DQ523226, EF125176, and EF125177.

## Abbreviations

Aldedh: Aldehyde dehydrogenase
DEAB: 4-Diethylaminobenzaldehyde
PCNA: proliferating cell nuclear antigen
RA: retinoic acid
Raldh: retinaldehyde dehydrogenase
RAR: retinoic acid receptor
RNAi: RNA interference
siRNAs: small interfering RNAs
WBR: whole body regeneration
